# Microscopic Analysis of the *Tupanvirus* Cycle in *Vermamoeba vermiformis*

**DOI:** 10.3389/fmicb.2019.00671

**Published:** 2019-04-03

**Authors:** Lorena C. F. Silva, Rodrigo Araújo Lima Rodrigues, Graziele Pereira Oliveira, Fabio Pio Dornas, Bernard La Scola, Erna G. Kroon, Jônatas S. Abrahão

**Affiliations:** ^1^Laboratório de Vírus, Departamento de Microbiologia, Instituto de Ciências Biológicas, Universidade Federal de Minas Gerais, Belo Horizonte, Brazil; ^2^Faculdade de Ciências Básicas e da Saúde, Departamento de Farmácia, Universidade Federal do Vale do Jequitinhonha e Mucuri, Diamantina, Brazil; ^3^Faculté de Médecine, Aix-Marseille Université, Marseille, France

**Keywords:** *Tupanvirus*, viral characterization, viral cycle, giant viruses, *Vermamoeba vermiformis*

## Abstract

Since *Acanthamoeba polyphaga mimivirus* (APMV) was identified in 2003, several other giant viruses of amoebae have been isolated, highlighting the uniqueness of this group. In this context, the tupanviruses were recently isolated from extreme environments in Brazil, presenting virions with an outstanding tailed structure and genomes containing the most complete set of translation genes of the virosphere. Unlike other giant viruses of amoebae, tupanviruses present a broad host range, being able to replicate not only in *Acanthamoeba* sp. but also in other amoebae, such as *Vermamoeba vermiformis*, a widespread, free-living organism. Although the *Tupanvirus* cycle in *A. castellanii* has been analyzed, there are no studies concerning the replication of tupanviruses in other host cells. Here, we present an in-depth microscopic study of the replication cycle of *Tupanvirus* in *V. vermiformis*. Our results reveal that *Tupanvirus* can enter *V. vermiformis* and generate new particles with similar morphology to when infecting *A. castellanii* cells. *Tupanvirus* establishes a well-delimited electron-dense viral factory in *V. vermiformis*, surrounded by lamellar structures, which appears different when compared with different *A. castellanii* cells. Moreover, viral morphogenesis occurs entirely in the host cytoplasm within the viral factory, from where complete particles, including the capsid and tail, are sprouted. Some of these particles have larger tails, which we named “supertupans.” Finally, we observed the formation of defective particles, presenting abnormalities of the tail and/or capsid. Taken together, the data presented here contribute to a better understanding of the biology of tupanviruses in previously unexplored host cells.

## Introduction

Since the isolation of *Acanthamoeba polyphaga mimivirus* (APMV) in the early 2000s, giant viruses have been arousing interest due to their structural, biological, and genomic complexity (La Scola et al., 2003; Colson et al., 2017). Since then, questions have been raised about the relationship of these viruses to their hosts, their evolution, and their position in the microbial world. After about 15 years of study, several other giant viruses of amoebae were isolated, such as the marseilleviruses, pandoraviruses, and pithoviruses, among others, contributing further knowledge about the diversity of this group (Colson et al., 2017). Many other interesting and unusual viruses can be spread across a wide range of environments, so the discovery and characterization of these viruses is still a promising field and a major challenge (Colson et al., 2017).

In 2015, the prospection of giant viruses from 17 samples from soda lakes and oceanic soil sediments collected in Brazil was performed, resulting in the isolation of two new viral isolates, named *Tupanvirus* soda lake (TPVsl) and *Tupanvirus* deep ocean, which are able to replicate in amoebae of different genera, such as *Acanthamoeba* and *Vermamoeba*, among others (Abrahão et al., 2018). Due to their genetic and phylogenetic characteristics, tupanviruses are proposed to be members of the family Mimiviridae, constituting a new genus “*Tupanvirus*” (Abrahão et al., 2018; Rodrigues et al., 2018). The biological characterization of *Tupanvirus* strains showed a peculiar structure. A capsid similar to that of a *Mimivirus* with the stargate portal on one side and surrounded by fibrils (Zauberman et al., 2008; Abrahão et al., 2018). However, the presence of a cylindrical tail attached to the capsid in the isolates, which can extend their sizes to more than 2 μm, seems to be the distinguishing feature of *Tupanvirus* particles compared with other giant viruses described until now (Abrahão et al., 2018). Mimiviruses attracted attention due to the presence of a large, icosahedral capsid associated with fibrils; pandoraviruses, cedratviruses, and pithoviruses show an ovoid morphology, are very large viruses, and have apical pores; however, in none of these viruses was there any structure resembling that of a tail, which is only found in tupanviruses (La Scola et al., 2003; Philippe et al., 2013; Legendre et al., 2014; Abrahão et al., 2018).

To date, the replication cycle of a *Tupanvirus* strain, TPVsl, has been analyzed in *A. castellanii* by electron microscopy, among other techniques (Abrahão et al., 2018). The analyses showed that the particles bind to the surface of the amoeba and penetrate the cell, likely by a phagocytic process. The stargate opens, and the inner capsid and tail membranes merge with the phagosomal membrane, releasing the genome into the cell cytoplasm. A viral factory of the volcano type is formed, wherein the genome replication and morphogenesis of new particles occur, as described for other mimiviruses (Suzan-Monti et al., 2007; Abrahão et al., 2018). The tail of the particle is supposedly attached to the capsid after its formation and closure, although there is no clear evidence about this step of *Tupanvirus* morphogenesis (Abrahão et al., 2018). In late stages of the cycle, the amoebic cytoplasm is filled with several viral particles, followed by cell lysis and particle release (Abrahão et al., 2018).

As tupanviruses were the first giant amoeba viruses that demonstrated this ability to replicate in protozoa belonging to different genera, this study aimed to analyze in detail the replication cycle of TPVsl in *V. vermiformis* to elucidate and compare the steps of its replication cycle with those already evidenced in *A. castellanii* and other aspects that still remain unclear.

## Materials and Methods

### Virus Preparation and Cells

*Tupanvirus* soda lake (TPVsl) was isolated from a soda lake sample from the Pantanal region in Brazil and was produced and purified as previously described (Abrahão et al., 2018). Briefly, *A. castellanii* (ATCC 30010) cells were grown in 75 cm^2^ cell culture flasks (Nunc, United States) in peptone–yeast extract–glucose (PYG) medium (Visvesvara and Balamuth, 1975) supplemented with 25 mg/mL fungizone (Amphotericin B, Cristalia, Brazil), 500 U/mL penicillin, and 50 mg/mL gentamicin (Schering-Plough, Brazil). After reaching confluence, the amoebae were infected at a multiplicity of infection (m.o.i) of 0.1 and incubated at 32°C until cytopathic effects (CPE) were observed. Supernatants from the infected amoebae were collected and filtered through a 0.8 μm filter to remove cell debris. The viruses were purified by centrifugation through a sucrose cushion (22%), suspended in phosphate-buffered saline (PBS), and stored at -80°C.

### Asynchronous Cycle of TPVsl in *V. vermiformis* and Transmission Electron Microscopy (TEM)

To investigate the asynchronous cycle of TPVsl in *V. vermiformis* cells (ATCC 20237), 25 cm^2^ cell culture flasks with 5 × 10^6^ of *V. vermiformis* in 10 mL of PYG medium were infected with TPVsl at a m.o.i. of 0.1 and incubated at 32°C for 36 h. After the period of infection, the cells were collected and submitted to three cycles of freezing (-80°C)/ thawing (25°C) for cell lysis and virus release. Samples were then clarified for total particles counting in Neubauer Chamber and for titration. The viral titer was determined using the TCID_50_ (tissue culture infective dose) method that was calculated using the Reed and Muench (1938) method in 96-well plates with 4 × 10^4^ amoebae per well. The rest of the cells were prepared for microscopy assays. For this, the infected *V. vermiformis* cells were collected, pelleted by centrifugation at 1500 *g* for 10 min, and fixed in microcentrifuge tubes with 1 mL of 2.5% glutaraldehyde solution in 0.1 M sodium phosphate buffer pH 7,4 for 1 h at room temperature. The samples were then washed three times with 0.1 M sodium phosphate buffer, post-fixed with 2% osmium tetroxide, and embedded in Epon resin. Ultrathin sections were then analyzed under TEM (Spirit BioTWIN FEI, 120 kV) at the Center of Microscopy of UFMG.

### Scanning Electron Microscopy

For analysis under scanning electron microscopy (SEM), the infected *V. vermiformis* cells were collected after 24–36 h of infection, lysed by freezing/thawing and pelleted by centrifugation at 1500 *g* for 10 min. After, they were added to round glass coverslips covered with poly-L-lysine, and fixed with 2.5% glutaraldehyde solution in 0.1 M cacodylate buffer pH 7,4 for 1 h at room temperature. The samples were then washed three times with 0.1 M cacodylate buffer and post-fixed with 1.0% osmium tetroxide for 1 h at room temperature. After a second fixation, the samples were washed three times with 0.1 M cacodylate buffer and immersed in 0.1% tannic acid for 20 min. The samples were then washed in cacodylate buffer and dehydrated by serial passage in ethanol solutions at concentrations ranging from 35 to 100%. Samples were subjected to critical point drying using CO_2_, placed in stubs and metallized with a 5-nm gold layer. The analyses were completed using SEM (FEG Quanta 200 FEI) at the Center of Microscopy of UFMG.

### TPVsl One-Step-Growth-Curve in *V. vermiformis*

To get one-step-growth-curve of TPV in *V. vermiformis*, 25-cm^2^ cell culture flasks with 5 × 10^6^ cells of *V. vermiformis* in 10 mL of PYG medium were infected with TPVsl at M.O.I. of 10 and incubated at 32°C. At different time points, the flasks were observed by light microscope to monitor the evolution of the CPE. Moreover, cells were collected and used for titration by TCID_50_ as described above.

## Results

### The Early Steps of *Tupanvirus* Infection in *Vermamoeba vermiformis*

**FIGURE 1:**
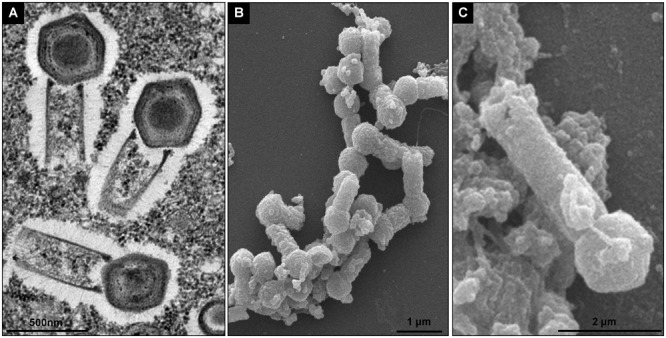
*Tupanvirus* soda lake particle. **(A)** Mature particle of Tupanvirus soda lake (TPVsl) in *V. vermiformis* under transmission electron microscopy (TEM). **(B)** Mature particle of TPVsl in *V. vermiformis* under scanning microscopy. Is it possible note the peculiar TPVsl morphology, with the tail attached to a *Mimivirus*-like capsid. **(C)** “Supertupan” in *V. vermiformis* under scanning electron microscopy.

To evaluate the replication profile of TPVs1 in *V. vermiformis*, asynchronous infections were performed, and the infected cells were prepared for electron microscopy analyses. Our first images revealed that the TPV particles in *Vermamoeba* cells acquire the same structure that was observed in *Acanthamoeba* cells (Figure 1) as described by Abrahão et al. (2018). Tupanviruses present a capsid of about 450 nm, similar to that of mimiviruses, including a stargate region in one of the vertexes and multiple layers, including an electron-dense structure inside the capsid, indicative of a lipid membrane. Attached to that capsid basis, there is a cylindrical tail approximately 450 nm in diameter and 550 nm in length, which increase the size of the virus, a unique feature displayed by these viruses (Figure 1). The complete viruses are approximately 1.2 μm, although some particles can be longer, reaching over 2.0 μm due to variation in tail size. We termed these larger particles “supertupans” (Figure 1C). Curiously, these enormous particles were observed recurrently in both our TEM and SEM preparations of infected *V. vermiformis* cells.

**FIGURE 2:**
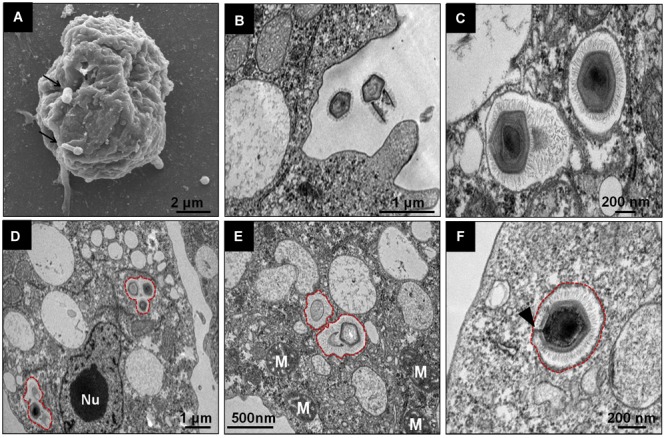
Initial steps of the replication cycle of *Tupanvirus* soda lake in *V. vermiformis*. **(A)** Detail of TPVsl particles attached to a *V. vermiformis* cell. **(B)** Amoebae emit pseudopodia to encompass viral particles that are internalized through phagocytosis. **(C–E)** Details of viral particles that remain within phagosomes (red outlines). The cell nucleus remains apparent/electrodense, and the cytoplasm presents several empty vacuoles. **(F)** During the uncoating step, the stargate opens, followed by membrane fusion. The viral capsid, indicated by the black arrow, releases the genome. M, mitochondria; Nu, nucleus.

Regarding the initial steps of the *Tupanvirus* cycle in *V. vermiformis*, the particles attach themselves to the surface of the host cell and penetrate the cell, most likely through phagocytosis, as amoebic pseudopods encompassing viruses close to their surface are observed (Figure 2A,B). Inside the amoeba cytoplasm, viruses stay within phagosomes, normally with one particle per phagosome, although multiple viruses can enter the host cell simultaneously, resulting in more than one particle inside a phagosome (Figure 2C–E). In these initial steps, we could observe the amoeba nucleus clearly without apparent changes, with the nucleolus highly evident (Figure 2D). Furthermore, mitochondria and several vacuoles were observed around the internalized viruses (Figure 2E). After, the stargate opened, and the viral capsid inner membrane fused with the phagosomes membrane, culminating with the release of the genome into the host cytoplasm (Figure 2F).

### Analysis of the *Tupanvirus* Viral Factory in *Vermamoeba vermiformis*

**FIGURE 3:**
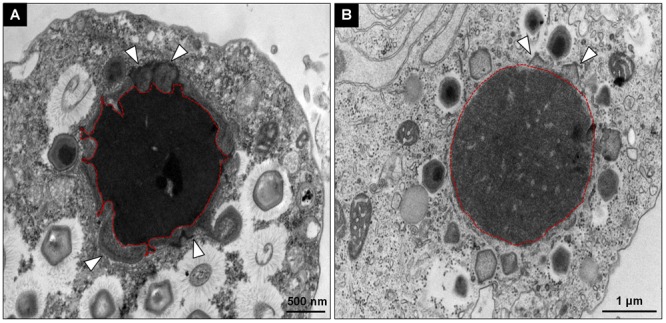
Comparison between the TPVsl viral factory (VF) in *V. vermiformis* and *A. castellanii*. **(A)** Mature VF in *V. vermiformis.* **(B)** Mature VF in *A. castellanii.* It is possible to note that the VF in the *Vermamoeba* on the left is electron-dense and has an irregular border in the periphery (red), from which sprouts the structures to form new viral particles. The budding of viral capsids is highlighted by the white arrows.

**FIGURE 4:**
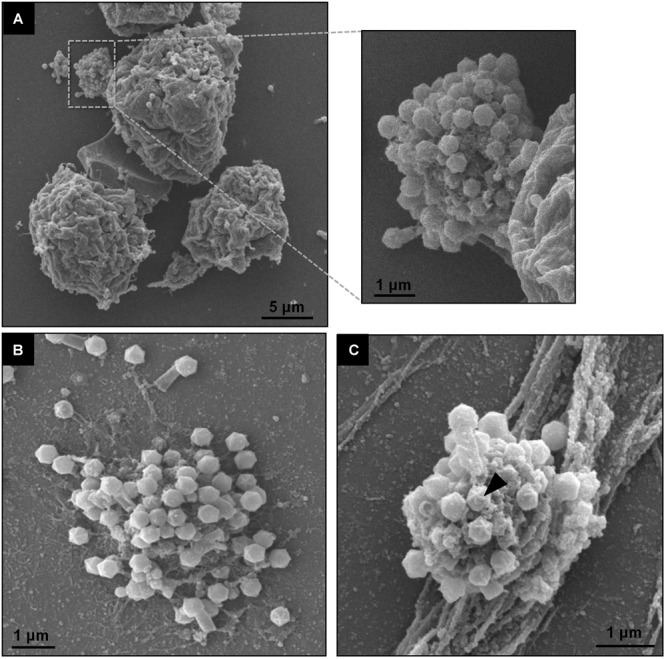
TPVsl viral factory by scanning microscopy. **(A)** Details of a mature VF involved in viral morphogenesis, including tail attachment. **(B,C)** Isolated VFs releasing viral particles. The stargate in a capsid is indicated by the black arrow.

**FIGURE 5:**
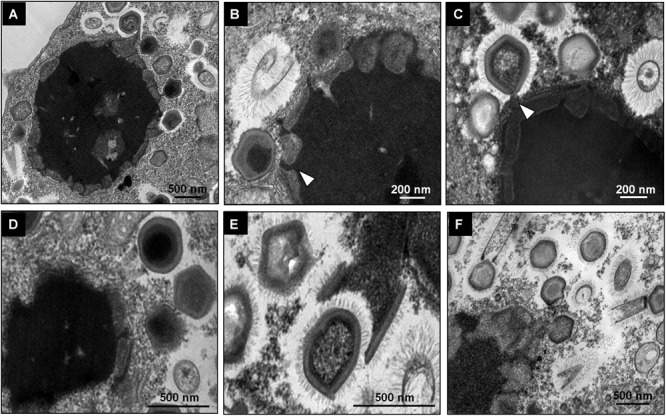
Morphogenesis of *Tupanvirus* particles. **(A)** Overview of a mature VF and the budding of the structures that compose the viral progeny through its periphery. **(B)** Genome acquisition (shown by the arrows) by a capsid without fibrils. **(C)** Genome acquisition by a capsid with fibrils. **(D–F)** Details of the sprouting of viral capsids and tails through the VF.

Giant viruses usually establish delimited regions in the hosts’ cytoplasm, named viral factories (VF) (Kuznetsov et al., 2013; Mutsafi et al., 2014; Andrade et al., 2017). Tupanviruses are no exception, as previously demonstrated upon infection of *A. castellanii* cells (Abrahão et al., 2018). According to our observations, TPVsl also establish a VF in the host cytoplasm when infecting *V. vermiformis* cells. After the early steps of infection, we observed the generation of a VF, a structure well delimited in the host’s cell cytoplasm (Figure 3A). The VF formed upon infection of *V. vermiformis* has a peculiar appearance (Figure 3A,B). Its margin is more delimited and irregular than in *A. castellanii*, evidencing a lamellar aspect of the VF in its mature stage (Figure 3A). The structure seems to be formed by several layers that expand in an way analogous to that of crescents described for poxviruses and marseilleviruses from where the viral structures sprout (Maruri-Avidal et al., 2011; Andrade et al., 2017). It is worthy of note that the VF of giant viruses is the region wherein the viral genome is replicated, and new particles are assembled (Kuznetsov et al., 2013; Mutsafi et al., 2014; Andrade et al., 2017). For that reason, several particles are expected to be found in these regions. This is valid for tupanviruses, since dozens of particles were observed in different SEM images (Figure 4). The particles appeared to be partially assembled, composed of a capsid and tail (Figure 4A), but fibrils were probably absent, since the stargate structure could be observed easily protruding in many particles, indicating that the particles, and the VF undergoes different levels of maturation (Figure 4B,C). At later stages of viral infection, once the VF is fully established, viral capsids are assembled, and the genome is incorporated at the periphery of the VF (Figure 5A–C). This event can occur before or after fibril acquisition, thus events are not likely to occur in chronological order (Figure 5D,E). In contrast to mimiviruses, it is likely that no particular area for fibril acquisition is formed during *Tupanvirus* VF maturation (Andrade et al., 2017). The viral tails apparently attach to the capsid immediately after genome incorporation and sprout from the VF along with the capsid, forming complete virions (Figure 5F).

### The Final Step of the TPVsl Cycle in *V. vermiformis* Is Associated With Defective Particle Release

**FIGURE 6:**
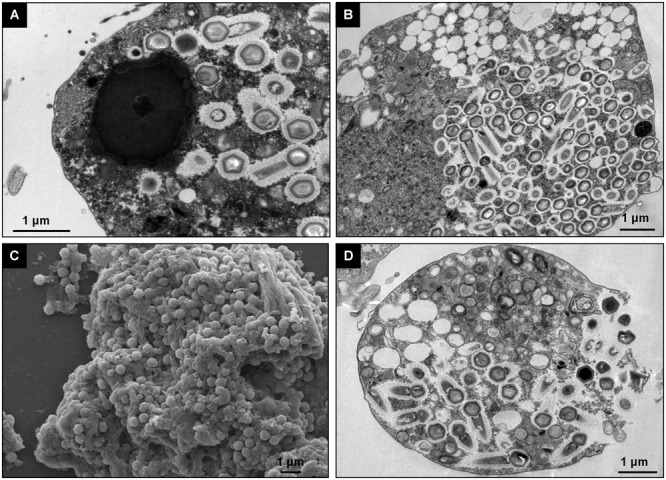
Final steps of the replication cycle of *Tupanvirus* in *V. vermiformis*. **(A)** VF in the mature stage releasing mature viral particles shown by TEM. **(B,C)** Amoebae filled with mature viral particles shown by transmission **(B)** and scanning **(C)** electron microscopy. **(D)** Amoeba cell filled with new viral particles under lysis shown by TEM.

**FIGURE 7:**
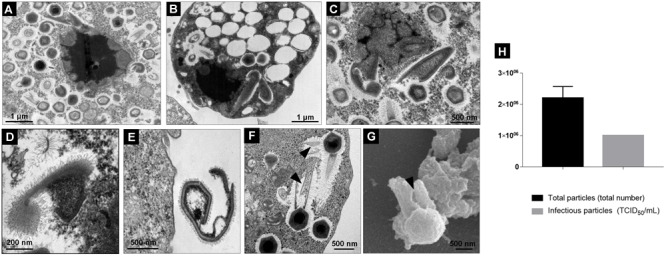
Defective particles of *Tupanvirus* formed during its cycle in *V. vermiformis*. **(A–C)** VF in the last step of maturation releasing mature and defective particles shown by TEM. **(D,E)** Details of defective particles shown by TEM. **(F)** Details of “supertupans” with defective tails (indicated by the black arrow) shown by TEM. **(G)** A defective tail of TPVsl show by scanning microscopy. **(H)** Proportion of total particles and infectious particles during the asynchronous cycle of TPVsl in *V. vermiformis.*

During the final step of the replication cycle, we observed a large increase in the number of typical TPVsl particles filling the cytoplasm, i.e., particles presenting a tailed capsid covered by fibrils and a size of approximately 1.2 μm (Figure 6A–C). Viral progeny formed by mature and complete particles accumulated in the amoebae cytoplasm and their release was mediated by cell lysis (Figure 6D). However, our analysis showed that this step is also associated with a high proportion of defective particles in *V. vermiformis* cells. Many images have shown that in some amoebae, the VF in its final stage presents a differentiated aspect: it is smaller, becomes less electron-dense, and loses its lamellar aspect (Figure 7A–C). This seems to be closely related to the budding of abnormal structures forming abnormal particles in the cytoplasm. In our analysis, we observed defective capsids without the expected pseudo-icosahedral symmetry and also not completely closed or surrounded by fibrils (Figure 7A–E). Furthermore, at this step we also observed defectives supertupans. Long tails are commonly noticed, and sometimes the cylindrical shape is replaced by undefined forms (Figure 7F). This process occurs in amoebae with final-stage mature VF. The comparative analysis of total particles and titrated particles obtained at the end of the asynchronous cycle showed that the number of total particles is about two times higher in relation to the infectious particles (Figure 7H).

### Characterization of CPE and Evolution of Viral Titer During Synchronous Infection

In order to characterize CPE triggered by *Tupanvirus* in *V. vermiformis*, that cells were infected at an m.o.i. of 10 and observed at up to 72 h.p.i. We observed that the formation of the CPE seems be slower in *V. vermiformis* than to that previously observed in *A. castellanii* (Abrahão et al., 2018). We observed that TPV induces in *V. vermiformis* cell rounding and early cluster formation, the typical “bunches” formed by TPV in amoeba (Oliveira et al., 2019), being visible only around 12 h.p.i., being most evident at 16 and 24 h.p.i. At 36–72 h p.i., we observed bunches disaggregation and lysis (Supplementary Figure S1A). One-step-growth-curve analysis revealed eclipse phase around 4 h.p.i. At 36 h.p.i., TPV title increases approximately 1 log (Supplementary Figure S1B) if compared to eclipse phase (4 h.p.i.), we observed titer increased about 3 log at 36 h.p.i.

## Discussion

Tupanviruses were isolated from extreme environments in Brazil and showed unprecedented characteristics, including the ability to replicate in different genera of protozoa (Abrahão et al., 2018). Our data suggest that TPV cycle in *V. vermiformis* is slower and less productive than TPV replication in *A. castellanii* (Oliveira et al., 2019; Supplementary Figure S1). The reason why we observe a delay in the evolution of TPV CPE in *V. vermiformis* requires more investigation as well. A similar profile was observed in the early phase of the replication cycle of TPVsl in *V. vermiformis* in relation to that which occurs in *A. castellanii*, with viral attachment to the amoeba surface and entry through phagocytosis (Abrahão et al., 2018). It is possible that tupanviruses attach to host cells by interaction of their fibrils with different glycans present on the cell surface, in a similar way to that observed for mimiviruses, although its composition remains to be elucidated (Rodrigues et al., 2015). The strategy of penetration by phagocytosis has recurrently been assumed for different giant viruses of amoebae, considering the size of the viral particles (larger than 500 nm) and the phagotrophic nature of amoebae (Suzan-Monti et al., 2007; Abrahão et al., 2014). However, it has been suggested that particles from smaller amoebae viruses such as marseilleviruses (approximately 250 nm) would not use this strategy but would use the other endocytic pathway or penetrate through phagocytosis when forming vesicles containing a large number of viral particles (Arantes et al., 2016). The phagocytic strategy for penetration was biologically demonstrated for APMV and *Cedratvirus getuliensis* by the use of pharmacological inhibitors of the phagocytosis process, demonstrating a considerable decrease in viral particle incorporation and replication success (Andrade et al., 2017; Silva et al., 2018). By observation of several TEM images, we suggest that this same strategy is adopted by TPVsl (Figure 2A,B), although other mechanisms, such as macropinocytosis, cannot be discarded for the moment.

The replication cycle appears to be entirely cytoplasmic, with the establishment of a well-defined VF, as previously reported for other related large DNA viruses (Mutsafi et al., 2010, 2014; Kuznetsov et al., 2013; Andrade et al., 2017). On the other hand, pandoraviruses are amoeba viruses that have a replication cycle involving the host nucleus in some way, due to the lack of genes essential for DNA replication in its genome, even though a large VF is observed (Philippe et al., 2013; Andrade et al., 2018). In this context, we noticed a difference in the aspect of the VF on the two amoeba cells infected by TPVsl (Figure 3). However, this characteristic should be observed with caution. This may be due to some particular property of this amoeba, including how it reacts to TEM preparation. And also, because asynchronous cycle was used, it is possible that the differences in the VF reflect different stages of the viral morphogenesis. Several studies with other amoeba giant viruses have demonstrated this close relationship between VF and morphogenesis (Suzan-Monti et al., 2007; Mutsafi et al., 2010, 2014; Kuznetsov et al., 2013; Andrade et al., 2017). For APMV, it was demonstrated that the assembly of capsids from increasing lamellar structures starts in the periphery of the VF, followed by membrane biogenesis and then genetic material packing on the opposite side of the stargate and simultaneous fibril acquisition by passage through a less electron-dense area surrounding the VF (Mutsafi et al., 2014; Andrade et al., 2017). For tupanviruses, we observed that the assembled capsid containing its various layers can be filled with DNA before or after fibril acquisition, since the VF of TPVsl does not present a delimited area for this event, neither in *V. vermiformis* nor in *A. castellanii*, in contrast to the observed for mimiviruses (Figure 5; Andrade et al., 2017).

Viral morphogenesis is a complex process during the replication cycle of a virus, in particular for the large DNA viruses, which involves the presence of many different and large structures (Moss, 2013; Andrade et al., 2017; Silva et al., 2018). Furthermore, some DNA viruses, such as herpesviruses, poxviruses, and mimiviruses, incorporate transcripts into their forming particles during this step (Raoult et al., 2004; Grossegesse et al., 2017). Recently, a next-generation sequencing (NGS) study showed that the content of transcripts incorporated by cowpox virus intracellular mature virion (IMV) in human cells (Hep-2) or murine cells (Rat-2), is not identical and thus may be due to host-specific incorporation (Grossegesse et al., 2017). Although no significant differences could be observed in the TPV cycle in *Acanthamoeba* and *Vermamoeba* concerning viral morphogenesis, we still cannot affirm that the content of transcripts and proteins in virions during their formation is the same in both cells. Further comparative studies involving genomics and proteomics would bring forward valuable information on this subject.

Viral progeny release is mediated by cell lysis in a similar way as previously demonstrated for other giant viruses (Figure 6; Abrahão et al., 2014). An interesting fact that drew attention at this step was the greatest presence of defective particles in *V. vermiformis* cytoplasm (Figure 7). This has already been verified for APMV in *A. castellanii* cells, suggesting that defective particles in giant viruses are not only formed in the presence of virophages but can also be an event associated with the normal replication cycle (La Scola et al., 2008; Andrade et al., 2017). Also, our data demonstrate that the proportion of total particles is about two-fold higher than the number of infectious particles after an asynchronous cycle, highlighting the presence of defective particles and corroborates with the observed images (Figure 7). The reason why there appears to be more defective TPV particles following infection in *Vermamoeba* requires further investigation. Considering that tupanviruses have a broad spectrum of hosts, in contrast to other giant amoeba viruses, it is possible that the level of adaptability of the viruses in different amoeba genera or species can influence this profile. In conclusion, the data presented here contribute to a better understanding of the biology of tupanviruses in *V. vermiformis*.

## Author Contributions

LS, RR, FD, and GO performed experiments. JA, BLS, and EK designed the study. All authors read and approved the final version of the manuscript.

## Conflict of Interest Statement

The authors declare that the research was conducted in the absence of any commercial or financial relationships that could be construed as a potential conflict of interest.

## Supplementary Material

FIGURE S1 Evolution of TPVsl cytopathic effect and infectious particles during the synchronous cycle. **(A)** *V. vermiformis* was infected with TPVsl using a high m.o.i. and visualized by light microscopy. We observed the formation of bunches after 12 h.p.i., that were disaggregated about 36 h.p.i. After this time, we observed lysis, but it was not total. The flasks were observed using the 100× objective on a light microscopy. **(B)** TPVsl one-step growth curve in *V. vermiformis* at an m.o.i. of 10. Error bars indicate standard deviation.
