# Supplementary figures and images for: Microscopic Analysis of the Tupanvirus Cycle in Vermamoeba vermiformis
Source: Front Microbiol. 2019 Apr 3;10:671. doi: 10.3389/fmicb.2019.00671 (PMC6456662; doi:10.3389/fmicb.2019.00671)

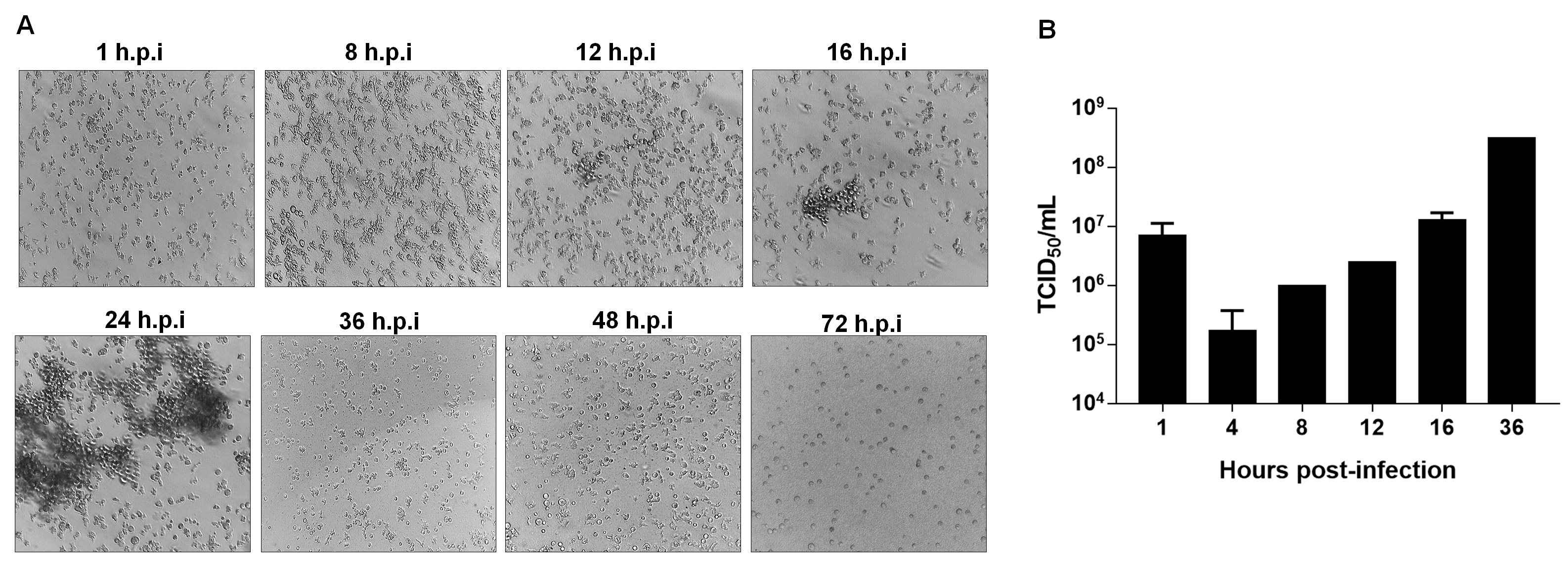

Supplement: FIGURE S1 — Evolution of TPVsl cytopathic effect and infectious particles during the synchronous cycle. (A) V. vermiformis was infected with TPVsl using a high m.o.i. and visualized by light microscopy. We observed the formation of bunches after 12 h.p.i., that were disaggregated about 36 h.p.i. After this time, we observed lysis, but it was not total. The flasks were observed using the 100× objective on a light microscopy. (B) TPVsl one-step growth curve in V. vermiformis at an m.o.i. of 10. Error bars indicate standard deviation. [file Image_1.TIF]
